# Effects of Eucalypt Plant Monoterpenes on Koala (*Phascolarctos Cinereus*) Cytokine Expression *In Vitro*

**DOI:** 10.1038/s41598-019-52713-5

**Published:** 2019-11-12

**Authors:** Caroline Marschner, Mark B. Krockenberger, Damien P. Higgins

**Affiliations:** Sydney School of Veterinary Science, Faculty of Science, The University of Sydney, Camperdown, 2006 NSW Australia

**Keywords:** Ecophysiology, Evolutionary ecology, Cytokines

## Abstract

Protective immunity is crucial for survival of any species, though the koala as a specialist feeder adapted to an exclusive diet of eucalypts that contain plant secondary metabolites of varying toxicity and of immunomodulatory property. Being constantly exposed to such dietary chemicals it raises the question of their immune effects in a specialist eucalypt feeder. This study demonstrates that natural levels of circulating eucalypt plant secondary metabolites have dose dependent *in vitro* effects on cytokine expression of koala peripheral blood mononuclear cells, suggesting a potential trade-off of reduced function in multiple arms of the immune system associated with koala’s use of its specialized dietary niche.

## Introduction

Widespread in the Australian landscape, eucalypts comprise an easily accessible food resource for some folivores, such as the koala *(Phascolarctos cinereus)*, the ringtail possum *(Pseudocheirus peregrinus) and* greater glider (*Petauroides volans*) that are able to exploit this dietary niche. The koala is able to utilise this resource exclusively for nutrition and shelter^1^ even though chemical defences render eucalypt leaves unpalatable, of low nutritional value, and even toxic. Tannin and lignin (up to 30% DM) bind valuable leaf proteins^2,3^ and cell wall carbohydrates^4^ in the gastrointestinal tract of herbivores. Most leaf fats are either indigestible waxes^2^ or toxic terpenoids.

Monoterpenes, an abundant group of terpenoids in eucalypts, are of small molecular weight and highly lipophilic, hence are readily absorbable from the gut wall of eucalypt folivores^5^, and enter the circulation^5–10^. In spite of the specialist strategy of leaf choice to avoid certain plant secondary metabolites (PSMs)^11–16^, specialist feeders remain exposed to high concentrations of multiple PSMs^7,9,17^. Those species rely on increased detoxification abilities to maintain their feeding rate^18^. A rapid metabolic clearance of eucalypt monoterpenes but also of pharmaceuticals was found in koalas *in vitro* and *in vivo*^19–26^. Extended oxidative (cytochrome P450, first phase) and conjugative (glucuronosyltransferase, second phase) detoxification pathways were identified in this species^27–31^. Metabolites of monoterpenes were found to be exclusively oxidized^6,32^.

The continuous intake of toxic eucalypt monoterpenes has always been considered as a dangerous trade. Toxicological data on eucalypt monoterpenes are limited but the acute oral toxicity of 1,8-cineole (the main eucalypt oil constituent) in rats is LD50: 2480 mg/kg^33^. In much lower concentrations, monoterpenes act as potent immunomodulatory components that can modify the expression of critical immune mediators. *In vivo* studies in mice have shown that monoterpenes s-limonene and myrcene significantly inhibit the production of the cytokines IFN-ɣ and IL-4 during LPS induced pleurisy^34^. The production of TNF-α, IL-1β, IL-4, IL-5, leukotriene and thromboxane^35^ was strongly suppressed by 1,8-cineole in *in vitro* assays of human lymphocytes and monocytes. The monoterpene p-cymene significantly reduces expression of the cytokines TNF-α, IL-1β and IL-6 in an *in-vivo* mouse model^36,37^. Other major eucalypt monoterpenes, such as terpinen-4-ol, *α*-terpineol significantly suppressed the production of TNF-α, IL-1β, IL-8 and IL-10 ^38^. These cytokines are key signalling glycoproteins that mediate communication between immune cells. Each individual immune-pathway, innate or adaptive (cell-mediated or humoral), relies on critically balanced interactions of particular cytokines^39,40^, which aim for an optimal immune response towards specific types of pathogens^41^ and are involved in all steps of acute and chronic inflammation^42,43^. Optimal balances are determined by host-pathogen co-evolution that occurs within the context of the host’s and pathogen’s environment. Thus, changes to host, pathogen or environment can perturb these balances and influence disease outcomes and impacts at individual or population levels.

Despite the known immunomodulatory actions of monoterpenes in experimental animal models, the immunological consequences of chronic exposure in a specialist eucalypt feeder, or increases to exposure that might be driven by restriced dietary choice, have not been investigated. Although the immune system of koalas is fundamentally similar to that of other mammalian species, it is unclear to what extent the koala’s immune cells have adapted to exposure to these compounds. Koalas‘ lymphatic anatomical structures are comparable to those of other species^44,45^ and animals show a cellular and humoral immune responses following vaccination^46^ or infection^47,48^. Circulating B:T cell ratios were found to be high^49^ or similar^50^ to that of other mammals and two subclasses of the immunoglobulins, IgG and IgM, have been identified in serum of koalas^51^. Koala peripheral blood leukocytes produce typical inflammatory cytokines in response to non-specific (mitogens)^52^ or specific (Chlamydia antigens) activation^53–55^. It is possible that either the koala immune cells have evolved resistance to the immunomodulatory effects of these substances or that some downregulation of immune system function has not been an evolutionary disadvantage relative to the benefits of access to this plentiful resource. It is important to understand if any evolutionary trade-offs exist as these may become unbalanced in the face of reduced dietary choice with habitat degradation, or introduction of new pathogens such as *Chlamydia* spp.

The aim of the present study was to analyse the effects of naturally encountered plasma concentrations of two major eucalypt monoterpenes, 1,8-cineole and p-cymene, on cytokine expression of koala peripheral blood mono-nuclear cells (PBMCs) in stimulation assays. The specific roles of inflammatory cytokines in pathological processes and their measurability make them a useful tool in immunological studies^56^. Primers for a whole set of koala immune genes, for CD4, CD8β, IFN-γ, IL-4, IL-6 and IL-10 ^52^ as well as IL-17A and TNF-α^54,55^, have been described previously. In the present study, cytokines promoting the Th1 (IFN-γ), Th2 (IL-10) and Th17 (IL-17A) pathways of the adaptive immune response and the innate immune response (IL-6, TNF-α) were assessed to test the hypothesis that levels of monoterpenes detected in the blood of koalas can reduce cytokine expression of koala immune cells and, therefore, potentially influence innate and adaptive immunity in this species.

## Methods and Materials

### Stimulation assays

The authors confirm that all methods were carried out in accordance with relevant guidelines and regulations. Experiments were approved by the Animal Ethics Committee of the University of Sydney (Protocol Nr.565) and by the New South Wales Government (Scientific licence SL101290).

Animals used for blood collection for stimulation assays were adult healthy koalas (3 male and 3 female) that are part of a captive population. Animals were kept under same conditions (females and males separated) and fed on the same eucalypt diet. All animals were chlamydia negative and being of NSW/Qld provenance, KoRV A positive.’

Blood was taken from koalas under manual restraint, transported on ice and lymphocyte stimulation assays were performed immediately based on established methods^49,52^, with the following minor modifications.

Isolated PBMCs were washed in PBS (1×) and seeded with culture media into 96 well plates in duplicates in concentrations of 5 × 10^5^ cells per well and exposed to half logarithmic serial dilutions of the eucalypt monoterpenes 1,8-cineole (eucalyptol 99%, analytical grade, C80601 ALDRICH, Sigma Aldrich) and p-cymene (99%, analytical grade C121452 ALDRICH, Sigma Aldrich).

Concentrations used in the current study (1,8-cineole: 194 nmol/l, 648 nmol/l, 1940 nmol/l, 6480 nmol/l, p-cymene 74.5 nmol/l, 223 nmol/l, 745 nmol/l, 2230nmol/l) simulated physiological blood levels (1,8-cineole: 194–6480 nmol/l, p-cymene: 74.5–2230 nmol/l) based on naturally occurring blood concentrations determined in another study using 54 free ranging and captive koalas (Marschner *et al*., 2019, unpublished data).

PBMCs were activated using the PMA/Ionomycin protocols as described in Maher, *et al*.^52^. Positive controls (activated, with no monoterpene exposure) and negative controls (not activated, no monoterpene exposure) were tested on each plate. Controls contained the same concentrations of carrier (0.45% ethanol) as cells exposed to monoterpene. RNA was extracted using the MagJET RNA Purification kit (K2731, Life Sciences Solutions ThermoFisher Scientific) and the “KingFisher™ Duo Prime Purification System” (ThermoFisher Scientific, Australia). Cytokine gene expression RTqPCR was conducted as previously described^52,57^.

### Lymphocyte proliferation assays

Lymphocyte viability was investigated to detect potential cytotoxic effects of 1.8- cineole and p-cymene in relevant concentrations. Based on validation in koalas by Lau^58^ the MTT assay was performed based on manufacturers recommendations (MTT Reagent, 10009591, Sapphire Bioscience Pty. Ltd, Australia). Intracellular NAD(P)H-dependent oxidoreductase enzymes reduce the applied tetrazolium (yellow dye, MTT) to formazan (purple dye). The formation of the formazan is dependent on cell metabolic activity and cell viability. The intensity of dye was read using a SpectraMax 250 micro-plate reader (Molecular Devices, Bio-Strategy Pty. Ltd. VIC 3061, Australia).

### Statistical methods

A linear mixed model was utilized, fitted with a REML, using GenStat (Version 18th, VSN international). Logarithmic values of cytokine up-regulation were used for analysis and model-based means were later back-transformed, including appropriate delta-method standard errors. Toxin type (1,8-cineole, p-cymene) and toxin concentration (1,8-cineole: 194 nmol/l, 648 nmol/l, 1940 nmol/l, 6480 nmol/l, p-cymene 74.5 nmol/l, 223 nmol/l, 745 nmol/l, 2230 nmol/l), as well as their interaction, were treated as fixed effects for each model. To take the positive control into account, additional terms for treatment (yes vs no) were included in the model, with the toxin, concentration and interaction terms nested within it. Additionally correlations between the cytokines were evaluated using two-sided test.

## Results

**Figure 1:**
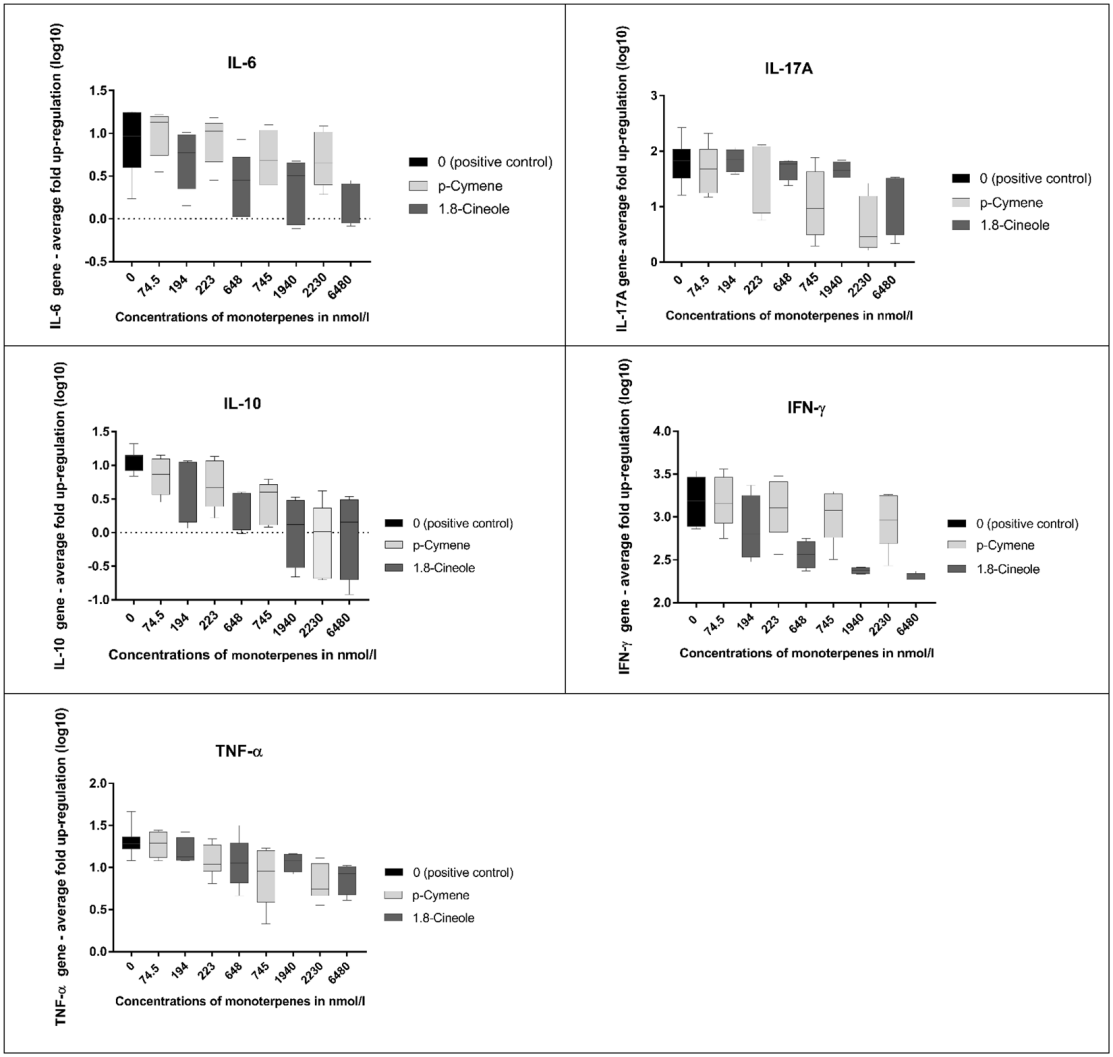
Illustrates the inhibitory effects of p-cymene and 1.8-cineole on up-regulation of cytokines in PBMCs of captive koalas (n = 6, 3 males:3 females). Graphs indicate the fold increase of up-regulation (including median with interquartiles) of IL-17, IL-6, IL-10, IFN-γ and TNF-α in PBMCs after stimulation using PMA/Ionomycin while exposed to different concentrations (in nmol/l) of the monoterpenes p-cymene (light grey) and 1,8-cineole (dark grey). Positive control is marked as column (black) at concentration zero, showing the up-regulation of IL-17A, IL-6, IL-10, IFN-γ and TNF-α of stimulated PBMCs without exposure to monoterpenes but only carrier. Error bars indicate standard errors.

No cytotoxic effects were found for monoterpene concentrations used in this study (*t-test*: *P*_1,8-cineole_ = 0.784, *P*_p-cymene_ = 0.979). For every cytokine there was a dose-dependent reduction in cytokine gene expression, regardless of the toxin (1.8- cineole/p-cymene, *P* < 0.001) (Fig. 1). There was a significant effect of toxin type on cytokine expression (all *P* ≤ 0.020) except for TNF-α (*P* = 0.92): expression of IL-17A gene was significantly more inhibited by p-cymene treatment compared to 1,8-cineole (*P* = 0.002) whereas 1,8-cineole treatment more effectively reduced expression of all other cytokines (IFN-γ *P* = < 0.001, IL-6 *P* = < 0.001, IL-10 *P* = 0.020). There were no significant difference between the shapes of the dose-response curves (on the logarithmic scale).

A two-sided *t-test* was used to test for significant correlations between cytokine expression. A significant correlation was found for TNF-α with several cytokines TNF-α/IL-6 (*P*_corr TNF-α/IL6_ = 0.012), TNF/IL-17A (*P*_corr TNF/IL-17A_ < 0.001) and TNF-α/IL-10 (*P*_corr TNF-α/IL-10_ < 0.001), but also for IL-6 and IL-10 (*P*_corr IL-6/IL-10_ < 0.001).

## Discussion

This study shows that koala PBMC are affected by eucalypt monoterpenes in a dose-dependent fashion, similarly to other species. Interestingly, results were comparable to those for humans^35^, in which concentrations (~9720 nmol/ml) of 1,8-cineol inhibited production of the cytokines TNF-alpha (by 92%), IL-1beta (by 84%), IL-4 (by 70%) and IL-5 (by 65%) in lymphocytes and, in monocytes, TNF-alpha (by 99%), IL-1beta (by 84%), IL-6 (by 76%), and IL-8 (by 65%). P-cymene dose-dependently down-regulated production of IL-6, TNF-α, and IL-1β l in mice^36,37^ and mixtures of relevant eucalypt monoterpenes significantly inhibited production of TNF-α, Il-6, TNF-α, IFN-γ, IL 10, IL-5 and IL-13 in human mononuclear cells^59^.

Other eucalypt monoterpenes have shown similar effects to those of 1.8-cineole and p-cymene. Limonene and β-myrcene reduced IFN-γ and IL-4 production of mononuclear cells significantly in non-toxic concentrations and when orally administered in mice prevented a delayed inflammatory reaction induced by LPS injection into the pleural cavity. A strong inhibition of cell migration of total leucocytes and mononuclear cells was found in this study^34^. Furthermore the monoterpenes terpinen-4-ol reduced significantly the production of TNF-α and Il-10 in human peripheral blood monocytes after LPS activation^38^.

Synergistic effects were observed in other studies when monoterpenes were administered in their natural occurring mixtures in form of essential oil extracts of plants which promoted even stronger immunosuppressive effects when compared to the application of single components^34,60^. Future studies could investigate synergistic effects of eucalypt monoterpenes in their naturally occurring composition in koala blood. Monoterpene profiles were published recently^61^.

In the present study p-cymene also had some evidence of stimulatory effects on cytokine expression in the lowest concentrations, though this finding was not consistent in all koalas. Such biphasic dose-responses (hormesis), whereby compounds have stimulatory effects in low doses but become inhibitory with increasing concentrations, are widespread in immunological studies^62^.

It appears that, similar to other species, koala PBMC function is affected by exposure to common eucalypt PSMs and yet, unlike other species, koala PBMC are regularly exposed to PSMs concentrations in order to exploit their dietary niche. This finding opens for consideration the novel perspective that the immunomodulatory effect of dietary toxins may be another important factor limiting the koalas use of particular habitats.

Dietary exposure to the immunomodulatory effects of PSMs might be advantageous if the low metabolic rate of the koala is considered, neutral, or may place constraints on ability to adapt to new pathogens or environmental conditions, making it of interest to explore these relationships further in future studies. Firstly it is possible that, along with lower body temperature and efficient pelt and tissue insulation^63,64^, PSM exposure has facilitated adaptation to a low energy food source, by limiting some energetically expensive aspects of the immune response. For maintenance, koalas only require 257 to 411KJ/kg^−0.75^ daily^65^, compared to other captive eutherian mammals with approximately 600 KJ/kg^−0.75^ ^66,67^. The initiation of an immune response to kill infectious pathogens increases resting metabolic rate^68,69^ and an induction of fever requires a 7–15% increase in energy requirements for each degree celsius of increase in body temperature^68^. Both the innate and adaptive immune responses are energetically expensive to either develop or maintain^70,71^. In view of a low energetic budget, immunological trade-offs are not unexpected^72^, would ultimately leave koalas less able to mount some immune responses and might involve one or more of several other alternative resistance or tolerance mechanisms.

The effects of PSMs on PMBC function and potentially immune function, may become more important under climate change or land use scenarios in which eucalypt PSMs increase^73,74^ or with introduction of novel pathogens. It appears likely that at least some strains of *Chlamydia pecorum*, a significant pathogen of koalas^75,76^ that requires specific immune pathways for elimination or control^77,78^, were brought into Australia with sheep and cattle during European settlement^79–81^ and, based on their impact on individuals and some populations, it appears feasible that koalas may not have the adaptive capacity to deal with this disease. Understanding these issues will require a better understanding of the co-evolutionary relationships between the koala’s pathogens and parasites, host responses and diet, and full investigation of this area would need to go beyond aspects of the host response explored in the current study. For example, in addition to more detailed examination of the immune system, the potential for interplay between PSM metabolism and defences based on depletion of pathogen nutrients, such as tryptophan, appear worth examining. Kynurenine pathways are effective modulators of pathogen tolerance by hosts through tryptophan depletion^82^. *Chlamydia pneumoniae*, which has a long evolutionary history with koalas and produces little disease^83^, lacks a specific tryptophan synthesis pathway and consequently is dependent on host tryptophan resources^84^, unlike *C*. *pecorum*, which exhibits a nearly complete biosynthetic pathway^85^. Kynurenines, products of tryptophan catabolism, induce Cytochrome P450 metabolism of xenobiotics such as PSMs through aryl hydrocarbon receptors (AhRs)^86^ and also allow the differentiation of regulatory T cells^87,88^ and restrict dendritic cell maturation^89,90^. Alternatively, monoterpenes have been shown to be antibacterial and antifungal^91–94^. There is currently no evidence they are protective in naturally occurring blood concentrations^95,96^ but act at least localy in the respiratory and oral tract through eucalypt oil ingestion^97–100^.

## Conclusions

A dose-dependent inhibition of cytokine expression *in vitro* was found in this study when koala PBMCs were activated in the presence of naturally occuring concentrations of two major eucalypt monoterpenes, 1,8-cineole and p-cymene. This finding opens a new area for future enquiry relevant to folivore and herbivore ecology and evolution: that of the role of dietary toxins in immunological trade-offs. Currently it is unclear the role that eucalypt monoterpenes play in koalas‘ immune resilience and fundamental research is needed in koala eco-immunology, to investigate the potential significance of any trade-offs to resilience of koala populations in response to changing environments and pathogens, particularly *Chlamydia* species.
